# A Significant Expansion of Our Understanding of the Composition of the Human Microbiome

**DOI:** 10.1128/mSystems.00010-19

**Published:** 2019-01-29

**Authors:** Sigal Leviatan, Eran Segal

**Affiliations:** a Department of Computer Science and Applied Mathematics, Weizmann Institute of Science, Rehovot, Israel; University of Chicago

**Keywords:** genome assembly, human microbiome

## Abstract

Shotgun sequencing of samples taken from the human microbiome often reveals only partial mapping of the sequenced metagenomic reads to existing reference genomes. Such partial mappability indicates that many genomes are missing in our reference genome set. This is particularly true for non-Western populations and for samples that do not originate from the gut. Pasolli et al. (E. Pasolli, F. Asnicar, S. Manara, M. Zolfo, et al., Cell, 2019, https://doi.org/10.1016/j.cell.2019.01.001) perform a grand effort to expand the reference set, and to better classify its members, revealing a wider pangenome of existing species as well as identifying new species of previously unknown taxonomic branches.

## Abstract (precis)

Shotgun sequencing of samples taken from the human microbiome often reveals only partial mapping of the sequenced metagenomic reads to existing reference genomes. Such partial mappability indicates that many genomes are missing in our reference genome set.

## COMMENTARY

The human microbiome is a complex ecology, consisting of tens of trillions of cells of a diverse community of microorganisms, mainly bacteria. But as of today, a large proportion of the metagenomics reads of any human microbiome sample is still unmappable to any known reference genome. This is true even for a typical gut microbiome sample of a Western individual but is even more pronounced for samples of non-Western individuals and for other body sites such as the skin. This nonmappability may stem from much wider pangenomes of the known species than we currently know of, or alternatively from new species that are yet unknown (1).

Pasolli et al. (2) performed a rigorous and well-documented effort to assemble high-quality genomes *de novo* from a wide range of studies that sequenced human microbiome cohorts. Altogether, the authors analyzed 9,316 samples obtained from 46 different data sets and originating from different populations, a variety of body sites, and multiple age groups. They supplemented these samples with a new cohort of 112 stool samples that they sequenced from the non-Westernized population of Madagascar. They then performed metagenome assembly of each sample separately and applied quality control measures to each assembled genome, controlling for completeness, contamination, and strain homogeneity. At the end of this process, the quality of their assembled genomes was on par with current reference genome sets such as the NCBI database.

**FIG 1:**
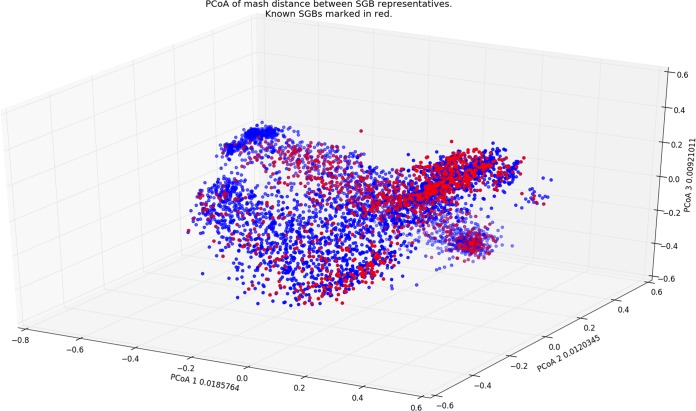
First 3 principal components of a PCoA transformation on the distances between the representatives of 4,390 SGB groups of assembled genomes. Representatives with a previously known assembly (from NCBI) are colored in red, and newly assembled representatives are colored blue, highlighting the greatly expanded set of genomes provided in this work.

These assemblies were then clustered by sequence similarity measures, together with a large set of reference genomes from the NCBI database. This allows defining thresholds of species-, genus-, and family-level similarities and led to the definition of 4,930 single-genome bins (SGBs) of human microbiome origin (many other reference genomes in the NCBI do not appear in the human microbiome). Remarkably, of the 4,930 SGBs, less than a quarter contain previously assembled reference genomes, and a fifth originate from entirely unexplored families (Fig. 1). A phylogenetic map, constructed for all these genomes, highlights entirely unexplored areas of the taxonomic tree.

Mapping new microbiome samples to this new set of 4,930 representatives of these SGBs (one representative per SGB, based on quality control measures) raised the mappability of a Western gut sample by an average of over 20% and doubled the mappability of many non-Western gut samples (from around 40%) to a level comparable to that of Western samples.

Pasolli et al. (2) also annotate the reconstructed genomes and are able to assign 230 million (230M) genes at UniRef90 (UniProt reference clusters of 90% similarity sequence identity to and 80% overlap with the longest protein sequence of the cluster) and another 38M at UniRef50. As expected, assembled groups with known species have more of their genes assigned to a UniRef (up to >90%) than those of any known genus or of known family alone. The lowest levels of gene assignments to UniRefs are for genes coming from assemblies which were not classified to any known family (down to 22% of marked genes being assigned to UniRef clusters).

On the pangenomic front, this work allows better insights into variation at the subspecies level which itself may affect the host (3). This approach to building a pangenome is also much less biased than the current existence of a large set of reference genomes. As an example of the utility of the approach of Pasolli et al. (2), the number of assembled variants of Prevotella copri, which is among the most abundant bacteria in the human gut, was expanded from a few genomes to more than a thousand, opening the door to further studies of the subspecies-level variance of *P. copri* between individuals and across populations. Obviously, no matter how many assemblies we gather, we will never be able to include the entire pangenome of a species, and species are ever evolving and expanding their pangenome. Indeed, no pangenome approached saturation even given the amount of sequence included in this study. However, the understanding of these intraspecies variations allows the study of phenotypic differences which might have to do with the differences between them (clear examples being differences between pathogenic and nonpathogenic intraspecies variants [4]).

At higher-level taxonomies, this work assembles and clusters new species, some of which are quite distant from any previously known species. For example, seven new SGBs that are phylogenetically located between *Faecalibacterium* and *Ruminococcus* were assembled, including some SGBs that are highly prevalent in the human microbiome. Interestingly, some of the differences between and inside these new SGBs have to do with differences between Westernized and non-Westernized populations, such as different pathways for vitamin B_12_ production, or gene clusters devoted to fatty acid biosynthesis and galactose metabolism.

Overall, this work represents an important expansion of our understanding of the human microbiome and a great resource and methodology that can readily be applied to additional diverse cohorts and data sets.
